# Predictors of elevated capillary blood glucose in overweight railway French employees: a cross-sectional analysis

**DOI:** 10.1186/s12889-018-5384-y

**Published:** 2018-04-16

**Authors:** Emminarie Luisiana Lucas Garcia, David Debensason, Loïc Capron, Antoine Flahault, Jeanine Pommier

**Affiliations:** 1 SNCF, Optim’Services - Services Médicaux, 4 rue André Campra CS 20012, 93212 La Plaine Saint-Denis Cedex, France; 2 Univ Rennes, EHESP, CNRS, ARENES-UMR 6051, F-35000 Rennes, France; 3 CPRP SNCF, Échelon National du Contrôle Médical, Marseille, France; 4 Institute of Global Health, Faculty of Medicine, University of Geneva, Geneva, Switzerland

**Keywords:** Elevated capillary blood glucose, Type 2 diabetes, Overweight, Body mass index, Employees, Workplace

## Abstract

**Background:**

Hyperglycaemia is a risk factor of cardiovascular disease and a high risk state for progression to type 2 diabetes. Moreover, overweight, defined as a body mass index (BMI) between 25 and 29.9 kg/m^2^, increases the risk of diabetes. Information about the feasibility of measuring, during routine occupational health examinations, predictors of elevated capillary blood glucose in overweight individuals is scarce. This study aims to identify factors that are associated with elevated capillary blood glucose and can be routinely measured in French overweight employees to develop targeted preventive strategies in the workplace.

**Methods:**

Cross-sectional study based on data collected during a workplace health promotion programme of the French National Railways Company (SNCF) from January 2011 to March 2015. A self-administered questionnaire was completed by overweight volunteers during the routine occupational health examination. Data collected included health, anthropometric, sociodemographic, occupational, and lifestyle characteristics. Elevated capillary blood glucose was defined as capillary blood glucose equal to or higher than 7 mmol/L. Multivariate logistic regression analysis was used to examine factors associated with elevated capillary blood glucose and results were described with odds ratios (OR) and 95% confidence intervals (CI).

**Results:**

The analysis concerned 2248 overweight employees (mean age: 43 years) with complete data (total population: 7724). The prevalence of elevated capillary blood glucose was 20.0%. In the multivariate analysis, significant predictors of elevated capillary blood glucose were: male sex (OR 1.66, 95% CI 1.21–2.28), age ≥ 50 years (OR 1.61, 95% CI 1.01–2.55), high blood pressure (OR 1.35, 95% CI 1.07–1.69), and daily intake of sugary food (OR 1.53, 95% CI 1.17–2.00). No association with occupational characteristics (work schedule, job seniority, professional grade, and job sector) was found possibly due to lack of statistical power.

**Conclusions:**

Our findings provide information for setting up specific diabetes prevention strategies in the workplace. Overweight men, aged 50 and older, with high blood pressure and daily sugary food intake should be considered for capillary blood glucose measurements during their occupational medical surveillance. Hypertension screening and management as well as health policy measures to target sugary food consumption could be included in workplace prevention strategies.

**Electronic supplementary material:**

The online version of this article (10.1186/s12889-018-5384-y) contains supplementary material, which is available to authorized users.

## Background

Diabetes affects approximately 422 million adults worldwide, among whom 300 million working-age adults (20–64 years) [1, 2]. Type 2 diabetes has a negative impact on the working population’s employment and productivity by reducing the ability to work [3]. Indeed, diabetes contributes to work disability, early retirement, unemployment, and premature death [4–7]. Type 2 diabetes also represents an important cause of sickness absence with a growing economic burden for employers [8, 9].

Type 2 diabetes is a chronic disease characterised by elevated levels of blood glucose as a result of pancreatic cell dysfunction or insulin resistance [10]. Frequently, diabetes remains undetected for many years because of the slow onset and progression of symptoms. During its prolonged asymptomatic phase, elevated blood glucose leads to the development of long-term chronic and invalidating complications, such as visual impairment, renal failure, coronary heart disease, stroke, neuropathy and peripheral vascular disease [11]. These complications may contribute to premature mortality and will have a negative impact on the quality of life. Moreover, hyperglycaemia is defined as a condition characterised by abnormally high level of blood glucose and it is recognised as a risk state in the progression for type 2 diabetes and a risk factor for other chronic conditions, such as cardiovascular disease (even below the threshold for diabetes diagnosis) [12].

Currently, a large proportion of adults with type 2 diabetes are undiagnosed and this represents a major public health issue [11]. Therefore, there is an urgent need to find strategies to improve the early identification of individuals at high risk of diabetes because the management of modifiable risk factors could stop or delay its appearance. Moreover, if started early, blood glucose level management with specific measures, such as changes in lifestyle behaviours and medications, can delay the progression of complications [13–15].

Many different risk factors contribute to increase the risk of diabetes, such as overweight and obesity, physical inactivity, unhealthy diet, smoking, hypertension, and also environmental and socioeconomic factors [12, 15, 16]. Moreover, several studies have shown that chronic conditions, such as obstructive sleep apnoea, are associated with higher incidence of type 2 diabetes [17, 18]. Occupational characteristics also have been related to type 2 diabetes. For instance, shift work, where eating and sleep patterns are significantly modified, seems to contribute to insulin resistance, and has been associated with higher risk of diabetes [19, 20]. Similarly, long working hours and occupational stress have been associated with diabetes risk [21–23]. Other studies reported an association between occupational class and the risk of diabetes, suggesting a higher risk for people who belong to the lower occupational classes [24].

Workplace has been identified as an ideal setting for disease prevention and health promotion strategies [25–27]. Screening initiatives carried out in the workplace during routine occupational health examinations can easily target the whole working population. Moreover, these initiatives could be particularly useful to reach specific populations, such as disadvantaged individuals, people with obesity, physically inactive individuals or with poor dietary habits, and smokers, who usually do not participate in health screening programmes carried out in primary care settings [28, 29].

Overweight (body mass index, BMI, between 25 and 29.9 kg/m^2^) is strongly associated with the risk of diabetes [30]. In France, the prevalence of type 2 diabetes is estimated at 6% and being treated for diabetes is three times more frequent among overweight adults compared with individuals with a BMI ≤ 25 kg/m^2^ [31, 32]. However, despite the number of scientific publications about the determinants of type 2 diabetes, information about the feasibility of measuring, during routine occupational health examinations, predictors of elevated capillary blood glucose in overweight individuals is still scarce.

This study on overweight employees from the French National Railways Company (SNCF) aimed to identify factors (health, anthropometric, sociodemographic, occupational and lifestyle characteristics) that are associated with elevated capillary blood glucose and that can be routinely measured in order to develop targeted preventive strategies in the workplace setting.

## Methods

### Population and study design

This cross-sectional study was based on data collected from overweight employees (BMI 25–29.9 kg/m^2^) who accepted to participate in a health screening during their occupational health examination as a part of a workplace health promotion programme carried out at the SNCF, between January 2011 and March 2015. SNCF is a national public-sector company and therefore, its employees cover a large diversity of occupations (train drivers, manual workers, administrative staff, health professionals, lawyers, etc.) classified in seven job sectors by the company: freight, infrastructure, material (maintenance, distribution), traction (train operators), transportation (station and on-board personnel), security and administrative staff. During the health screening, anthropometric measurements (weight, height, waist circumference, BMI) and health data (capillary blood glucose and blood pressure) were measured and collected by trained health professionals using standardised methods (i.e., same methods/equipment in all medical centres). Participants were asked to complete a self-administered questionnaire on sociodemographic, occupational and lifestyle characteristics (diet and physical activity), and the risk of sleep apnoea. Individuals with BMI ≥ 30 kg/m^2^ were referred to a specialist and were excluded from the study. A total of 7724 overweight employees agreed to take part in the health screening. After exclusion of participants with missing data, the study population consisted of 2248 overweight employees for whom complete data were available. All participants gave their written informed consent. The study protocol and data collection were approved by the Data Protection Officer of the French National Railways Company (Ref. D14.022.V.M00).

### Data collection

#### Outcome variable

Capillary blood glucose was based on measurements obtained by fingerprick and using a calibrated glucometer (BeneCheck, model BK-G-M001). Capillary blood glucose measurement is recommended as an alternative option in settings where venous plasma glucose measurement is not available or feasible for diabetes screening [21]. The outcome of interest, elevated capillary blood glucose, was defined on the basis of a threshold for random plasma glucose ≥ 7 mmol/L [33]. Capillary blood glucose was then categorised in normal (< 7 mmol/L) and elevated (≥ 7 mmol/L).

#### Sociodemographic and occupational characteristics

The self-administered questionnaire included sociodemographic information (sex, age, and marital status). Occupational characteristics included job seniority, which was divided in four categories (less than 10 years, 11–20 years, 21–30 years and ≥ 30 years), and work schedule (day only, night only, shift work, and rotating shift work). Moreover, the employees’ professional grade (low, intermediate and high, according to the company three-level scale) was also recorded, as well as the occupation, according to the main job sectors of the company previously described in the population and study design section.

#### Anthropometric and health data

Height (cm), weight (kg) and waist circumference (cm) were measured by trained occupational health nurses during the occupational health examination. The waist circumference was measured in the horizontal plane of the superior border of the iliac crest with the individual in standing position after a full expiration. Waist circumference was dichotomised in normal (< 88 cm for women and < 102 cm for men) and elevated (≥ 88 cm for women and ≥ 102 for men) [34, 35]. BMI was calculated as weight (kilograms) divided by the square of height (metres) and included in the analysis in two categories (BMI > 27.5 kg/m^2^ and BMI ≤ 27.5 kg/m^2^) according to the additional cut-off points proposed by the World Health Organization (WHO) [36, 37]. Blood pressure was assessed using an electronic upper-arm sphygmomanometer with the subject in seated position after 5 min of rest. Blood pressure was then categorised in normal (systolic blood pressure < 140 mmHg and diastolic blood pressure < 90 mmHg) and high (systolic blood pressure ≥ 140 mmHg and/or diastolic blood pressure ≥ 90 mmHg) according to the WHO recommended cut-off values [38–40].

#### Lifestyle characteristics

Lifestyle characteristics included information on diet and physical activity. Participants were questioned about their dietary habits during a working day: intake frequency of specific foods (fruits and vegetables, cereals and starchy carbohydrates, dairy products, meat and poultry, eggs, fish, ready-to-eat food and sugary food), addition of salt to meals at the table, and consumption of sugary drinks and alcoholic beverages.

Information on physical activity was collected using an adapted version of the General Practice Physical Activity Questionnaire (GPPAQ) translated from English to French. The GPPAQ assesses the physical activity in adults in the context of primary care services using a four level-physical activity index (active, moderately active, moderately inactive and inactive) [41].

#### Risk level of sleep apnoea

The Berlin sleep questionnaire that includes ten items covering three different domains (snoring, daytime sleepiness and risk factors) was used to evaluate the risk level of sleep apnoea (low and high risk) [42].

### Statistical analysis

All statistical analyses were conducted using the SAS software version 9.3 (SAS Institute, Cary, North Carolina, USA). Descriptive analysis was performed and frequencies and percentages were reported for categorical variables. Percentages were compared using the chi-square test. Logistic regression analysis was used to determine predictors of elevated capillary blood glucose in terms of odds ratios (OR) and 95% confidence intervals (CI). All variables associated with elevated capillary blood glucose with *p* < 0.10 in the univariate logistic regression analysis were retained for the multivariate analysis. For multivariate logistic regression analysis, a stepwise variable selection method was used. A *p* value less than 0.05 was considered statistically significant.

Additionally, the impact of missing data was assessed by performing all the analyses in the whole study population (i.e., including all the individuals with missing data, *n* = 7724). Sensitivity analyses were also conducted to assess the impact of missing data. The multiple imputation method for categorical variables based on a cumulative logistic regression model was applied using PROC MI in SAS. The multiple imputation model was constructed based on the BMI category and all the variables retained in the multivariate logistic regression model from the complete-case analysis. Five imputed datasets were created and each dataset was analysed. Then, results were combined using PROC MIANALYZE in SAS.

## Results

### Population characteristics

The study population included 1832 men (81.5%) and 416 women (18.5%) with a mean age of 43 years (age range: 19–62). Table 1 shows the characteristics of the study participants. The prevalence of elevated capillary blood glucose was 20.0% (*n* = 450), 21.7% and 12.7% in men and women respectively. A large majority of the respondents belonged to the low professional grade (42.1%, *n* = 946), followed by the intermediate grade (30.9%, *n* = 695), and the high grade (27.0%, *n* = 607). About two thirds of participants had a day work schedule (*n* = 1359, 60.5%). High blood pressure was detected in 28.6% (*n* = 642) of participants, and high current risk level of sleep apnoea in 16.9% (*n* = 380). A body mass index between 25.0 and 27.4 kg/m^2^ was found in 49.4% (*n* = 1111) of respondents, and elevated waist circumference in 35.8% (*n* = 804). Concerning the diet characteristics, daily intake of sugary foods (once per day or more) was reported by 23.7% (*n* = 534) of participants. Overall, 30% (*n* = 674) of respondents consumed sugary drinks once or twice per day and 32.7% (*n* = 735) drunk alcoholic beverages ≥ 3 times/week. Finally, 31.8% of participants (*n* = 715) were classified in the inactive category for physical activity and only 22.2% (*n* = 499) in the active category.

**Table 1:** Characteristics of the study population

Characteristics	Total	Capillary blood glucose		
		Normal (< 7 mmol/L) *n* (%)	Elevated (≥ 7 mmol/L) *n* (%)	*p* value
	(*n* = 2248)	1798 (80.0)	450 (20.0)	
Sex				
Women	416 (18.5)	363 (87.3)	53 (12.7)	< 0.001
Men	1832 (81.5)	1435 (78.3)	397 (21.7)	
Age (years)				
< 30	163 (7.2)	137 (84.0)	26 (16.0)	< 0.001
31–39	657 (29.2)	562 (85.5)	95 (14.5)	
40–49	716 (31.9)	561 (78.3)	155 (21.7)	
≥ 50	712 (31.7)	538 (75 .6)	174 (24.4)	
Marital status				
Married/living with a partner	1916 (85.2)	1535 (80.1)	381 (19.9)	0.71
Single	332 (14.8)	263 (79.2)	69 (20.8)	
Professional grade				
Low	946 (42.1)	742 (78.4)	204 (21.6)	0.28
Intermediate	695 (30.9)	561 (80.7)	134 (19.3)	
High	607 (27.0)	495 (81.6)	112 (18.4)	
Job sector				
Administrative staff	281 (12.5)	231 (82.2)	50 (17.8)	0.76
Freight	117 (5.2)	96 (82.0)	21 (18.0)	
Infrastructure	785 (34.9)	625 (79.6)	160 (20.4)	
Material	340 (15.1)	263 (75.4)	77 (22.6)	
Traction	238 (10.6)	195 (81.9)	43 (18.1)	
Transportation	450 (20.0)	358 (79.6)	92 (20.4)	
Security	37 (1.7)	30 (81.1)	7 (18.9)	
Job seniority (years)				
≤ 10	465 (20.7)	391 (84.1)	74 (15.9)	< 0.01
11–20	704 (31.3)	577 (82.0)	127 (18.0)	
21–30	596 (26.5)	465 (78.0)	131 (22.0)	
≥ 30	483 (21.5)	365 (75.6)	118 (24.4)	
Work schedule				
Day only	1359 (60.5)	1087 (80.0)	272 (20.0)	0.48
Shift work	517 (23.0)	422 (81.6)	95 (18.4)	
Rotating shift work	338 (15.0)	264 (78.1)	74 (21.9)	
Night only	34 (1.5)	25 (73.5)	9 (26.5)	
BMI (kg/m^2^)				
25 to 27.4	1111 (49.4)	906 (81.6)	205 (18.4)	0.07
27.5 to 29.9	1137 (50.6)	892 (78.5)	245 (21.6)	
Waist circumference				
Normal	1444 (64.2)	1160 (80.3)	284 (19.7)	0.58
Elevated	804 (35.8)	638 (79.4)	166 (20.7)	
Blood pressure				
Normal	1606 (71.4)	1318 (82.1)	288 (17.9)	< 0.001
High	642 (28.6)	480 (74.8)	162 (25.2)	
Risk of sleep apnoea				
Low	1868 (83.1)	1505 (80.6)	363 (19.4)	0.12
High	380 (16.9)	293 (77.1)	87 (22.9)	
Physical activity				
Inactive	715 (31.8)	577 (80.7)	138 (19.3)	0.72
Moderately inactive	468 (20.8)	367 (78.4)	101 (21.6)	
Moderately active	566 (25.2)	450 (79.5)	116 (20.5)	
Active	499 (22.2)	404 (81.0)	95 (19.0)	
Diet				
Cereals/bread				
Never or less than 3 times per week	172 (7.6)	135 (78.5)	37 (21.5)	0.69
3 to 6 times per week	350 (15.6)	287 (82.0)	63 (18.0)	
1 or 2 times per day	1119 (49.8)	896 (80.1)	223 (19.9)	
At least, three times per day	607 (27.0)	480 (79.1)	127 (20.9)	
Rice/pasta				
Never or less than 3 times per week	282 (12.5)	224 (79.4)	58 (20.6)	0.79
3 to 6 times per week	1350 (60.1)	1083 (80.2)	267 (19.8)	
1 or 2 times per day	599 (26.6)	479 (80.0)	120 (20.0)	
At least, three times per day	17 (0.8)	12 (70.6)	5 (29.4)	
Dairy products				
Never or less than 3 times per week	216 (9.6)	172 (79.6)	44 (20.4)	0.65
3 to 6 times per week	530 (23.6)	427 (80.6)	103 (19.4)	
1 or 2 times per day	1254 (55.8)	994 (79.3)	260 (20.7)	
At least, three times per day	248 (11.0)	205 (82.7)	43 (17.3)	
Fruits and vegetables				
Never or less than 3 times per week	192 (8.5)	149 (77.6)	43 (22.4)	0.24
3 to 6 times per week	923 (41.1)	725 (78.6)	198 (21.4)	
1 or 2 times per day	943 (41.9)	765 (81.1)	178 (18.9)	
At least, three times per day	190 (8.5)	159 (83.7)	31 (16.3)	
Meat and poultry, eggs and fish				
Never or less than 3 times per week	125 (5.5)	93 (74.4)	32 (25.6)	0.33
3 to 6 times per week	822 (36.6)	652 (79.3)	170 (20.7)	
1 or 2 times per day	1261 (56.1)	1021 (81.0)	240 (19.0)	
At least, three times per day	40 (1.8)	32 (80.0)	8 (20.0)	
Ready-to-eat food				
Never or less than 3 times per week	1797 (79.9)	1437 (80.0)	360 (20.0)	0.12
3 to 6 times per week	395 (17.6)	322 (81.5)	73 (18.5)	
1 or 2 times per day	56 (2.5)	39 (69.6)	17 (30.4)	
Sugary food				
Never or less than 3 times per week	874 (38.9)	719 (82.3)	155 (17.7)	< 0.05
3 to 6 times per week	840 (37.4)	674 (80.2)	166 (19.8)	
Once per day or more	534 (23.7)	405 (75.8)	129 (24.2)	
*You add to your meals…*				
Salt				
Never/sometimes	1316 (58.5)	1052 (79.9)	264 (20.1)	0.95
Often/always	932 (41.5)	746 (80.0)	186 (20.0)	
*You drink…*				
Sugary drinks				
Never or less than 3 times per week	718 (31.9)	590 (82.2)	128 (17.8)	< 0.05
3 to 6 times per week	458 (20.4)	367 (80.1)	91 (19.9)	
1 or 2 times per day	674 (30.0)	544 (80.7)	130 (19.3)	
At least, three times per day	398 (17.7)	297 (74.6)	101 (25.4)	
Alcoholic beverages				
Never or less than 3 times per week	1513 (67.3)	1231 (81.4)	282 (18.6)	< 0.05
3 times or more per week	735 (32.7)	567 (77.1)	168 (22.9)	

### Logistic regression analysis

The potential association of all the variables presented in Table 1 with the outcome variable (elevated capillary blood glucose) was assessed in the univariate logistic regression analysis (Table 2).

**Table 2:** Unadjusted and adjusted ORs for predictors of elevated capillary blood glucose in overweight employees

	Unadjusted ORs (95% CI)	*p* value	Adjusted ORs (95% CI)	*p* value
Variables				
Sex				
Women	1 (reference)		1 (reference)	
Men	1.89 (1.39–2.58)	< 0.001	1.66 (1.21–2.28)	< 0.01
Age (years)				
< 30	1 (reference)		1 (reference)	
31–39	0.89 (0.56–1.43)	0.63	0.89 (0.55–1.43)	0.62
40–49	1.46 (0.92–2.30)	0.11	1.42 (0.89–2.25)	0.14
≥ 50	1.70 (1.08–2.68)	< 0.05	1.61 (1.01–2.55)	< 0.05
Marital status				
Married/living with a partner	1 (reference)	0.70		
Single	1.06 (0.79–1.41)			
Professional grade				
Low	1 (reference)			
Intermediate	0.87 (0.68–1.11)	0.26		
High	0.82 (0.64–1.06)	0.14		
Job sector				
Administrative staff	1 (reference)			
Freight	1.01 (0.58–1.77)	0.97		
Infrastructure	1.18 (0.83–1.68)	0.35		
Material	1.35 (0.91–2.01)	0.14		
Traction	1.02 (0.65–1.60)	0.94		
Transportation	1.19 (0.81–1.74)	0.38		
Security	1.08 (0.45–2.59)	0.87		
Job seniority (years)				
≤ 10	1 (reference)			
11–20	1.16 (0.85–1.59)	0.35		
21–30	1.49 (1.09–2.04)	< 0.05		
≥ 30	1.71 (1.24–2.36)	< 0.01		
Work schedule				
Day only	1 (reference)			
Shift work	0.90 (0.69–1.17)	0.42		
Rotating shift work	1.12 (0.84–1.49)	0.44		
Night only	1.44 (0.66–3.12)	0.36		
BMI (kg/m^2^)				
25 to 27.4	1 (reference)			
27.5 to 29.9	1.21 (0.99–1.49)	0.07		
Waist circumference				
Normal	1 (reference)			
Elevated	1.06 (0.86–1.32)	0.58		
Blood pressure				
Normal	1 (reference)		1 (reference)	
High	1.54 (1.24–1.92)	< 0.001	1.35 (1.07–1.69)	< 0.05
Risk of sleep apnoea				
Low	1 (reference)			
High	1.23 (0.94–1.60)	0.12		
Physical activity				
Inactive	1 (reference)			
Moderately inactive	1.15 (0.86–1.54)	0.34		
Moderately active	1.08 (0.82–1.42)	0.59		
Active	0.98 (0.74–1.32)	0.91		
Diet				
Cereals/bread				
Never or less than 3 times per week	1 (reference)			
3 to 6 times per week	0.80 (0.51–1.26)	0.34		
1 or 2 times per day	0.90 (0.61–1.34)	0.63		
At least, three times per day	0.97 (0.64–1.45)	0.87		
Rice/pasta				
Never or less than 3 times per week	1 (reference)			
3 to 6 times per week	0.95 (0.69–1.31)	0.76		
1 or 2 times per day	0.97 (0.68–1.38)	0.85		
At least, three times per day	1.61 (0.55–4.76)	0.39		
Dairy products				
Never or less than 3 times per week	1 (reference)			
3 to 6 times per week	0.94 (0.64–1.40)	0.77		
1 or 2 times per day	1.02 (0.72–1.46)	0.90		
At least, three times per day	0.82 (0.51–1.31)	0.40		
Fruits and vegetables				
Never or less than 3 times per week	1 (reference)			
3 to 6 times per week	0.95 (0.65–1.38)	0.77		
1 or 2 times per day	0.81 (0.55–1.18)	0.26		
At least, three times per day	0.67 (0.41–1.13)	0.13		
Meat and poultry, eggs and fish				
Never or less than 3 times per week	1 (reference)			
3 to 6 times per week	0.76 (0.49–1.17)	0.21		
1 or 2 times per day	0.68 (0.45–1.04)	0.08		
At least, three times per day	0.73 (0.30–1.74)	0.47		
Ready-to-eat food				
Never or less than 3 times per week	1 (reference)			
3 to 6 times per week	0.90 (0.69–1.20)	0.48		
1 or 2 times per day	1.74 (0.97–3.11)	0.06		
Sugary food				
Never or less than 3 times per week	1 (reference)		1 (reference)	
3 to 6 times per week	1.14 (0.89–1.46)	0.28	1.20 (0.94–1.54)	0.14
Once per day or more	1.48 (1.14–1.92)	< 0.01	1.53 (1.17–2.00)	< 0.01
*You add to your meals…*				
Salt				
Never/sometimes	1 (reference)			
Often/always	0.99 (0.80–1.23)	0.95		
*You drink…*				
Sugary drinks				
Never or less than 3 times per week	1 (reference)			
3 to 6 times per week	1.14 (0.85–1.54)	0.38		
1 or 2 times per day	1.10 (0.84–1.44)	0.48		
At least, three times per day	1.57 (1.17–2.10)	< 0.01		
Alcoholic beverages				
Never or less than 3 times per week	1 (reference)			
3 times or more per week	1.29 (1.04–1.60)	< 0.05		

Overweight employees who were on night work (OR 1.44, 95% CI 0.66–3.12; *p* = 0.36) or rotating shift work (OR 1.12, 95% CI 0.84–1.49; *p* = 0.44) as well as those with elevated waist circumference (OR 1.06, 95% CI 0.86–1.32; *p* = 0.58), and high risk of sleep apnoea (OR 1.23, 95% CI 0.94–1.60; *p* = 0.12) were more likely to present elevated capillary blood glucose. However, these results did not reach statistical significance (Table 2). In addition, overweight employees with high professional grade (OR 0.82, 95% CI 0.64–1.06; *p* = 0.14), daily intake of dairy products (3 times per day) (OR 0.82, 95% CI 0.51–1.31; *p* = 0.40), and daily intake of fruits and vegetables (3 times per day) (OR 0.67, 95% CI 0.41–1.13; *p* = 0.13) were less likely to have elevated capillary blood glucose, but these results were not statistically significant.

On the other hand, sex, age, job seniority, BMI, blood pressure, consumption of meat, poultry, eggs and fish, ready-to-eat food, sugary foods intake, consumption of sugary drinks and alcoholic beverages were significantly associated with elevated capillary blood glucose (*p* < 0.10) (Table 2). All variables significantly associated with elevated capillary blood glucose in univariate analyses were retained for the multivariate logistic regression analysis using a stepwise variable selection method.

Multivariate analysis revealed that men (OR 1.66, 95% CI 1.21–2.28) and older employees (age ≥ 50 years: OR 1.61, 95% CI 1.01–2.55) had a higher likelihood of elevated capillary blood glucose. Similarly, individuals with high blood pressure were significantly more likely to have elevated capillary blood glucose than those with normal blood pressure (OR 1.35, 95% CI 1.07–1.69). Finally, participants reporting a daily sugary food intake (once per day or more) had a higher likelihood for elevated capillary blood glucose compared with participants reporting a sugary food intake of less than 3 times per week (OR 1.53, 95% CI 1.17–2.00). The adjusted ORs with 95% CI for elevated capillary blood glucose predictors in overweight employees are shown in Table 2.

In addition, the impact of the missing data was tested by performing all the analyses in the whole population (*n* = 7724). Similar results were obtained with the two datasets, with a similar proportion of people with elevated capillary blood glucose (17.7%, *n* = 1367/7724). Moreover, no significance change was observed when the analysis of the whole population (*n* = 7724) was restricted to the five variables selected in the multivariate analysis (Table 2). Actually, all the point estimates of the adjusted ORs were found within the confidence intervals presented in Table 2. Results are presented in Additional file 1: Table S1. Additionally, results obtained from analyses performed in the multiple imputed data showed that all the associations were in the same direction (see Additional file 2: Table S2).

## Discussion

The present study investigated the predictors of elevated capillary blood glucose in a sample of French overweight employees of a large public-sector company. To our knowledge, this is the first study carried out to investigate which factors proved to be measurable and associated with elevated capillary blood glucose among overweight individuals in order to develop targeted preventive strategies in the workplace setting in France. Our findings show that 20% (*n* = 450) of the overweight employees included in the study had elevated capillary blood glucose levels and this condition was significantly associated with male sex, older age (≥ 50 years), high blood pressure and regular sugary food consumption (once per day or more). In France, the prevalence of overweight is progressively increasing, and therefore these findings are particularly relevant in terms of diabetes prevention strategies in workplace settings, because of the deleterious impact of high blood glucose levels on health.

Our study reveals a higher likelihood of elevated capillary blood glucose among men. According with previous studies, men generally tend to have higher levels of blood glucose compared with women [43] and type 2 diabetes is more prevalent in men [1, 31]. Moreover, this finding is in line with a previous study carried out in the USA in which screening for impaired glucose tolerance using random blood glucose measurements showed higher risk of elevated blood glucose among men [33]. Our study also shows a higher likelihood of having elevated capillary blood glucose among ≥ 50-year-old participants, in agreement with previous studies on working populations that reported an increased diabetes prevalence with age among overweight individuals [44]. Moreover, alterations of the glucose metabolism and glucose intolerance are frequently observed during aging [45]. Additionally, the significant association between high blood pressure and elevated capillary blood glucose is in line with the existing literature indicating that hyperglycaemia can induce arterial damage and facilitate the development of atherosclerosis [46, 47]. Moreover, high blood glucose levels, but still below the threshold for diabetes diagnosis, are strongly correlated with mortality by stroke and ischemic heart disease [48], two conditions that imply arterial damage [49]. As diabetes and vascular disease share important pathophysiological pathways, these two conditions often affect the same individuals.

Substantial literature supports the association between unhealthy diet and chronic diseases, such as diabetes mellitus and hypertension [50]. Our findings highlight that regular sugary food intake (once or more per day) increases the probability of elevated capillary blood glucose among overweight individuals. Although a large number of studies have highlighted the controversial association between sugary food intake and risk of chronic diseases [51–53], other works showed that sugary food intake can be associated with diabetes through different pathways [54].

On the other hand, it has been suggested that regular consumption of fruits and vegetables and of dairy products is inversely associated with the risk of diabetes [55, 56]. Moreover, high intake of fruits/vegetables and consumption of dairy products have been related to better glycaemic control and lower incidence of hyperglycaemia, respectively [57–59]. The occupational characteristics (night work and rotating shift work) and the presence of chronic disorders, such as obstructive sleep apnoea, also have been linked to impaired glucose metabolism [19, 20, 60]. The presence of a social gradient has been also suggested; individuals with higher occupational class present a lower risk of hyperglycaemia than those individuals belonging to lower occupational class [61]. Our results, although not statistically significant, are in line with these previous findings. Due to the small sample size, our study may not have had sufficient power to detect statistically significant associations.

### Strengths and limitations

A major strength of this study is the diversity of factors included in the analysis: sociodemographic and occupational characteristics, health and anthropometric measurements, lifestyle characteristics (diet, physical activity), and current risk of sleep apnoea. Moreover, anthropometric and health data were measured and collected by trained occupational health professionals using standardised methods (i.e., same methods/equipment in all medical centres). However, our study has also some limitations. First, except for the anthropometric data and health information, all data were self-reported. This could have introduced a bias because participants tend to answer according to expected norms about physical activity and dietary characteristics [62, 63]. Moreover, underreporting about habitual food intake is more frequent in people with overweight and obesity than in individuals with normal body weight [64]. In addition, self-reported data are subject to recall bias. Second, capillary blood glucose measurement was used to identify elevated blood glucose among the study population. Although capillary sampling is accepted by the WHO, venous plasma glucose is the recommended standard method for blood glucose measurement [12]. Moreover, given the schedule of the routine occupational health examinations during the work day, most employees were not in a fasted state at the time of capillary blood glucose measurement. However, it has been shown that the performance of random blood glucose measurements is not affected by meals [33]. Third, the data included in the analysis concerned overweight volunteers who agreed to participate in the health screening programme and a large proportion of participants were excluded from the analysis because of missing data. This can limit the generalisation of our findings. It is possible that due to the small sample size, our analysis did not have enough statistical power to detect all significant associations. To control for possible bias, we performed all the analyses also in the whole sample, including participants with missing values (*n* = 7224), and we conducted sensitivity analyses using a multiple imputation model. Another limitation to take into account is the “healthy worker effect” because our study sample was based on data from workers who might probably have a better health status compared to the general population. Furthermore, blood pressure was measured only once and some participants could have been misclassified as having high blood pressure because of the stress or anxiety of visiting a doctor or due to the acute effect of caffeine taken before the occupational health examination [39, 65]. Similarly, some participants could have been classified as having elevated capillary blood glucose, although they had a transitory abnormal glucose metabolism due to psychological stress [66] or glucocorticoid treatment [67, 68]. Moreover, participants with a prior diabetes diagnosis were not excluded from the screening programme, and information regarding diabetes confirmation and follow-up was not available for individuals with elevated capillary blood glucose. Additionally, our study did not assess the effect of work stress or psychological stress that could be additional risk factors for impaired glucose metabolism [69, 70]. Finally, due to the cross-sectional design of our study, causal inferences could not be established.

### Relevance of findings for health professionals and policymakers

The findings of our study may have implications for occupational health practice and for corporate public health policy. First, the characterisation of predictors of elevated capillary blood glucose among overweight individuals may facilitate the detection of individuals at high risk for diabetes by occupational health professionals. Then, for such high-risk individuals, capillary blood glucose screening could be included in their regular assessment performed during the occupational health examination. Occupational health professionals could refer overweight individuals with elevated capillary blood glucose for diabetes confirmatory testing and for behavioural counselling on diet and physical activity. Moreover, blood pressure control and confirmatory diagnosis of hypertension should be incorporated into the routine management of this high-risk population. Regarding corporate public health policies, our findings support the need of a workplace environment that provides easy healthier choices for employees. Previous research has shown that environmental strategies can contribute to encourage and facilitate health behaviours by modifying the placement and/or the properties of stimuli or objects in a specific environment [71–74]. For instance, these interventions could be focused on improving the availability of healthy options in the workplace canteens and vending machines and reducing the availability of sugary foods. In addition, implementation of workplace health promotion programmes, including diet behaviour counselling for high-risk individuals, could contribute to maintain a healthy working population.

## Conclusion

Our findings show that male sex, age ≥ 50 years, high blood pressure and a self-reported daily sugary food intake were associated with elevated capillary blood glucose in our population of overweight employees. Selecting overweight employees helped restricting size of the target population prone to the campaign for health promotion and prevention. In France, the prevalence of overweight is continuously increasing; therefore, targeted screening strategies at the workplace to detect overweight individuals with elevated capillary blood glucose could contribute to the early identification of people with high risk of diabetes. This is particularly relevant for large companies that have their own occupational services because they could potentially play a major role in screening and prevention programmes. Moreover, among people with diabetes, elevated capillary blood glucose detection during the routine occupational health examination could help to improve glycaemic control and diabetes management. Our results suggest that some occupational characteristics (work schedule, job seniority, professional grade, and job sector) were not associated with elevated capillary blood glucose in overweight individuals probably due to lack of statistical power. Further research should be done to confirm and identify other potential predictors of elevated capillary blood glucose in overweight individuals in order to inform the development of specific prevention strategies in the workplace.

## Additional files

Additional file 1: **Table S1.** Adjusted ORs for predictors of elevated capillary blood glucose in the whole population (*n* = 7224). Description of data: **Table S1.** presents the results from the multivariate analysis in the whole population (*n* = 7224). Variables included in the model: sex, age, blood pressure, and sugary food. (DOCX 20 kb)

Additional file 2: **Table S2.** Multivariate logistic regression model using data from multiple imputation. Description of data: **Table S2.** presents the results obtained from sensitivity analyses (multiple imputation method) performed to assess the impact of missing data. (DOCX 18 kb)

## Abbreviations

BMI: Body mass index
CI: Confidence interval
GPPAQ: General Practice Physical Activity Questionnaire
h: Hour
kg: Kilogram
m: Metre
OR: Odds ratio
SNCF: French National Railways Company
WHO: World Health Organization

## References

[1] NCD Risk Factor Collaboration (NCD-RisC) (2016). Worldwide trends in diabetes since 1980: a pooled analysis of 751 population-based studies with 4.4 million participants. Lancet Lond Engl.

[2] International Diabetes Federation (2015). IDF diabetes atlas.

[3] Breton M-C, Guénette L, Amiche MA, Kayibanda J-F, Grégoire J-P, Moisan J (2013). Burden of diabetes on the ability to work: a systematic review. Diabetes Care.

[4] Virtanen M, Ervasti J, Mittendorfer-Rutz E, Tinghög P, Lallukka T, Kjeldgård L (2015). Trends of diagnosis-specific work disability after newly diagnosed diabetes: a 4-year Nationwide prospective cohort study. Diabetes Care.

[5] Kouwenhoven-Pasmooij TA, Burdorf A, Roos-Hesselink JW, Hunink MGM, Robroek SJW (2016). Cardiovascular disease, diabetes and early exit from paid employment in Europe; the impact of work-related factors. Int J Cardiol.

[6] Ervasti J, Virtanen M, Pentti J, Lallukka T, Tinghög P, Kjeldgard L (2015). Work disability before and after diabetes diagnosis: a nationwide population-based register study in Sweden. Am J Public Health.

[7] Lallukka T, Ervasti J, Mittendorfer-Rutz E, Tinghög P, Kjeldgård L, Pentti J (2016). The joint contribution of diabetes and work disability to premature death during working age: a population-based study in Sweden. Scand J Public Health.

[8] Asay GRB, Roy K, Lang JE, Payne RL, Howard DH (2016). Absenteeism and employer costs associated with chronic diseases and health risk factors in the US workforce. Prev Chronic Dis.

[9] Bishu KG, Gebregziabher M, Dismuke CE, Egede LE (2015). Quantifying the incremental and aggregate cost of missed workdays in adults with diabetes. J Gen Intern Med.

[10] Chatterjee S, Khunti K, Davies MJ (2017). Type 2 diabetes. Lancet Lond. Engl..

[11] Beagley J, Guariguata L, Weil C, Motala AA (2014). Global estimates of undiagnosed diabetes in adults. Diabetes Res Clin Pract.

[12] Roglic G (2016). World Health Organization, editors. Global report on diabetes.

[13] Merlotti C, Morabito A, Pontiroli AE (2014). Prevention of type 2 diabetes; a systematic review and meta-analysis of different intervention strategies. Diabetes Obes Metab.

[14] Diabetes Prevention Program Research Group (2015). Long-term effects of lifestyle intervention or metformin on diabetes development and microvascular complications over 15-year follow-up: the diabetes prevention program outcomes study. Lancet Diabetes Endocrinol.

[15] Ley SH, Hamdy O, Mohan V, Hu FB (2014). Prevention and management of type 2 diabetes: dietary components and nutritional strategies. Lancet Lond. Engl..

[16] Kolb H, Martin S (2017). Environmental/lifestyle factors in the pathogenesis and prevention of type 2 diabetes. BMC Med.

[17] Nagayoshi M, Punjabi NM, Selvin E, Pankow JS, Shahar E, Iso H (2016). Obstructive sleep apnea and incident type 2 diabetes. Sleep Med.

[18] Reutrakul S, Mokhlesi B (2017). Obstructive Sleep Apnea and Diabetes: A State of the Art Review. Chest.

[19] Gan Y, Yang C, Tong X, Sun H, Cong Y, Yin X (2015). Shift work and diabetes mellitus: a meta-analysis of observational studies. Occup Environ Med.

[20] Wang F, Zhang L, Zhang Y, Zhang B, He Y, Xie S (2014). Meta-analysis on night shift work and risk of metabolic syndrome. Obes Rev Off J Int Assoc Study Obes.

[21] Bannai A, Yoshioka E, Saijo Y, Sasaki S, Kishi R, Tamakoshi A (2016). The risk of developing diabetes in association with long working hours differs by shift work schedules. J Epidemiol.

[22] Li J, Jarczok MN, Loerbroks A, Schöllgen I, Siegrist J, Bosch JA (2013). Work stress is associated with diabetes and prediabetes: cross-sectional results from the MIPH industrial cohort studies. Int J Behav Med.

[23] Yu H, Liu J, Fan Y, Li C, Zhang L, Chen X (2016). Association between occupational stressors and type 2 diabetes among Chinese police officers: a 4-year follow-up study in Tianjin. China Int Arch Occup Environ Health.

[24] Hedén Stahl C, Novak M, Hansson P-O, Lappas G, Wilhelmsen L, Rosengren A (2014). Incidence of type 2 diabetes among occupational classes in Sweden: a 35-year follow-up cohort study in middle-aged men. Diabet Med J Br Diabet Assoc.

[25] Quintiliani L, Sattelmair J, Sorensen G. The workplace as a setting for interventions to improve diet and promote physical activity. Doc. Téc. Prep. Para El Even. Conjunto OMSForo Económico Mund. Sobre Prev. Las Enfermedades No Transm. En El Lugar Trab. Ginebra Organ. Mund. Salud [Internet]. 2007 [cited 2016 Feb 1]; Available from: http://www.who.int/entity/dietphysicalactivity/Quintiliani-workplace-as-setting.pdf.

[26] Hymel PA, Loeppke RR, Baase CM, Burton WN, Hartenbaum NP, Hudson TW (2011). Workplace health protection and promotion: a new pathway for a healthier--and safer--workforce. J Occup Environ Med.

[27] WHO | Workplace health promotion. WHO. [cited 2016 Dec 16]. Available from: http://www.who.int/occupational_health/topics/workplace/en/index1.html.

[28] Bjerregaard A-L, Maindal HT, Bruun NH, Sandbæk A (2017). Patterns of attendance to health checks in a municipality setting: the Danish ‘check your health preventive program.’. Prev Med Rep.

[29] Hoebel J, Starker A, Jordan S, Richter M, Lampert T (2014). Determinants of health check attendance in adults: findings from the cross-sectional German health update (GEDA) study. BMC Public Health.

[30] Ganz ML, Wintfeld N, Li Q, Alas V, Langer J, Hammer M (2014). The association of body mass index with the risk of type 2 diabetes: a case-control study nested in an electronic health records system in the United States. Diabetol Metab Syndr.

[31] Chevreul K, Berg Brigham K, Bouché C (2014). The burden and treatment of diabetes in France. Glob Health.

[32] INSERM, TNS Healthcare, Roche (2012). Enquête épidémiologique nationale sur le surpoids et l’obésité.

[33] Ziemer DC, Kolm P, Foster JK, Weintraub WS, Vaccarino V, Rhee MK (2008). Random plasma glucose in serendipitous screening for glucose intolerance: screening for impaired glucose tolerance study 2. J Gen Intern Med.

[34] Alberti KGMM, Zimmet P, Shaw J (2006). Metabolic syndrome--a new world-wide definition. A consensus statement from the international diabetes federation. Diabet. Med. J. Br. Diabet. Assoc..

[35] Expert Panel on Detection, Evaluation, and Treatment of High Blood Cholesterol in Adults (2001). Executive summary of the third report of the National Cholesterol Education Program (NCEP) expert panel on detection, evaluation, and treatment of high blood cholesterol in adults (adult treatment panel III). JAMA.

[36] Global BMI Mortality Collaboration (2016). Body-mass index and all-cause mortality: individual-participant-data meta-analysis of 239 prospective studies in four continents. Lancet Lond Engl.

[37] WHO : Global Database on Body Mass Index [Internet]. [cited 2013 Jul 23]. Available from: http://apps.who.int/bmi/index.jsp?introPage=intro_3.html.

[38] James PA, Oparil S, Carter BL, Cushman WC, Dennison-Himmelfarb C, Handler J (2014). 2014 evidence-based guideline for the management of high blood pressure in adults: report from the panel members appointed to the eighth joint National Committee (JNC 8). JAMA.

[39] Mancia G, Fagard R, Narkiewicz K, Redon J, Zanchetti A, Böhm M (2013). ESH/ESC guidelines for the management of arterial hypertension: the task force for the management of arterial hypertension of the European Society of Hypertension (ESH) and of the European Society of Cardiology (ESC). Eur Heart J.

[40] World Health Organization (2013). A global brief on hypertension. Silent killer, global public health crisis.

[41] Department of Health (2009). The general practice physical activity questionnaire: a screening tool to assess adult physical activity levels, within primary care.

[42] Netzer NC, Stoohs RA, Netzer CM, Clark K, Strohl KP (1999). Using the berlin questionnaire to identify patients at risk for the sleep apnea syndrome. Ann Intern Med.

[43] Tabák AG, Herder C, Rathmann W, Brunner EJ, Kivimäki M (2012). Prediabetes: a high-risk state for diabetes development. Lancet.

[44] Oberlinner C, Neumann SM, Ott MG, Zober A (2008). Screening for pre-diabetes and diabetes in the workplace. Occup Med Oxf Engl.

[45] Kalyani RR, Egan JM (2013). Diabetes and altered glucose metabolism with aging. Endocrinol Metab Clin N Am.

[46] Bornfeldt KE, Tabas I (2011). Insulin resistance, hyperglycemia, and atherosclerosis. Cell Metab.

[47] Paneni F, Beckman JA, Creager MA, Cosentino F (2013). Diabetes and vascular disease: pathophysiology, clinical consequences, and medical therapy: part I. Eur Heart J.

[48] Danaei G, Lawes CMM, Vander Hoorn S, Murray CJL, Ezzati M (2006). Global and regional mortality from ischaemic heart disease and stroke attributable to higher-than-optimum blood glucose concentration: comparative risk assessment. Lancet Lond. Engl..

[49] Beckman JA, Paneni F, Cosentino F, Creager MA (2013). Diabetes and vascular disease: pathophysiology, clinical consequences, and medical therapy: part II. Eur Heart J.

[50] Ezzati M, Riboli E (2013). Behavioral and dietary risk factors for noncommunicable diseases. N Engl J Med.

[51] Basu S, Yoffe P, Hills N, Lustig RH (2013). The relationship of sugar to population-level diabetes prevalence: an econometric analysis of repeated cross-sectional data. PLoS One.

[52] Macdonald IA (2016). A Review of recent evidence relating to sugars, insulin resistance and diabetes. Eur J Nutr.

[53] Rippe J, Angelopoulos T (2016). Relationship between added sugars consumption and chronic disease risk factors: current understanding. Nutrients.

[54] Johnson RK, Appel LJ, Brands M, Howard BV, Lefevre M, Lustig RH (2009). Dietary sugars intake and cardiovascular health: a scientific statement from the American Heart Association. Circulation.

[55] Wang P-Y, Fang J-C, Gao Z-H, Zhang C, Xie S-Y (2016). Higher intake of fruits, vegetables or their fiber reduces the risk of type 2 diabetes: a meta-analysis. J Diabetes Investig.

[56] Chen M, Sun Q, Giovannucci E, Mozaffarian D, Manson JE, Willett WC (2014). Dairy consumption and risk of type 2 diabetes: 3 cohorts of US adults and an updated meta-analysis. BMC Med.

[57] Carter P, Gray LJ, Talbot D, Morris DH, Khunti K, Davies MJ (2013). Fruit and vegetable intake and the association with glucose parameters: a cross-sectional analysis of the Let’s prevent diabetes study. Eur J Clin Nutr.

[58] Shin H, Yoon YS, Lee Y, Kim C, Oh SW (2013). Dairy product intake is inversely associated with metabolic syndrome in Korean adults: Anseong and Ansan cohort of the Korean genome and epidemiology study. J Korean Med Sci.

[59] Babio N, Becerra-Tomás N, Martínez-González MÁ, Corella D, Estruch R, Ros E (2015). Consumption of yogurt, low-fat milk, and other low-fat dairy products is associated with lower risk of metabolic syndrome incidence in an elderly Mediterranean population. J Nutr.

[60] Kim NH, Cho NH, Yun C-H, Lee SK, Yoon DW, Cho HJ (2013). Association of obstructive sleep apnea and glucose metabolism in subjects with or without obesity. Diabetes Care.

[61] Moody A, Cowley G, Ng Fat L, Mindell JS (2016). Social inequalities in prevalence of diagnosed and undiagnosed diabetes and impaired glucose regulation in participants in the health surveys for England series. BMJ Open.

[62] Miller TM, Abdel-Maksoud MF, Crane LA, Marcus AC, Byers TE (2008). Effects of social approval bias on self-reported fruit and vegetable consumption: a randomized controlled trial. Nutr J.

[63] Prince SA, Adamo KB, Hamel ME, Hardt J, Connor Gorber S, Tremblay MA (2008). Comparison of direct versus self-report measures for assessing physical activity in adults: a systematic review. Int J Behav Nutr Phys Act.

[64] Lissner L (2002). Measuring food intake in studies of obesity. Public Health Nutr.

[65] Corti R (2002). Coffee acutely increases sympathetic nerve activity and blood pressure independently of caffeine content: role of habitual versus nonhabitual drinking. Circulation.

[66] Williams ED, Magliano DJ, Tapp RJ, Oldenburg BF, Shaw JE (2013). Psychosocial stress predicts abnormal glucose metabolism: the Australian diabetes, obesity and lifestyle (AusDiab) study. Ann Behav Med Publ Soc Behav Med.

[67] Kauh E, Mixson L, Malice M-P, Mesens S, Ramael S, Burke J (2012). Prednisone affects inflammation, glucose tolerance, and bone turnover within hours of treatment in healthy individuals. Eur J Endocrinol.

[68] Rafacho A, Ortsater H, Nadal A, Quesada I (2014). Glucocorticoid treatment and endocrine pancreas function: implications for glucose homeostasis, insulin resistance and diabetes. J Endocrinol.

[69] Garbarino S, Magnavita N (2015). Work stress and metabolic syndrome in police officers. A prospective study. PLoS One.

[70] Bergmann N, Gyntelberg F, Faber J (2014). The appraisal of chronic stress and the development of the metabolic syndrome: a systematic review of prospective cohort studies. Endocr. Connect.

[71] Kahn-Marshall JL, Gallant MP (2012). Making healthy behaviors the easy choice for employees: a review of the literature on environmental and policy changes in worksite health promotion. Health Educ Behav.

[72] Quintiliani L, Poulsen S, Sorensen G (2010). Healthy eating strategies in the workplace. Int J Workplace Health Manag.

[73] Allan J, Querstret D, Banas K, de Bruin M. Environmental interventions for altering eating behaviours of employees in the workplace: a systematic review. . Obes. Rev. Off. J. Int. Assoc. Study Obes. 2017;18:214–226. 10.1111/obr.12470 27860169

[74] Hollands GJ, Shemilt I, Marteau TM, Jebb SA, Kelly MP, Nakamura R (2013). Altering micro-environments to change population health behaviour: towards an evidence base for choice architecture interventions. BMC Public Health.

