# Supplementary material for: Predictors of elevated capillary blood glucose in overweight railway French employees: a cross-sectional analysis
Source: BMC Public Health. 2018 Apr 16;18:507. doi: 10.1186/s12889-018-5384-y (PMC5902963; doi:10.1186/s12889-018-5384-y)
Supplement: Supplementary file 1 — Table S1. Adjusted ORs for predictors of elevated capillary blood glucose in the whole population (n = 7224). Description of data: Table S1. presents the results from the multivariate analysis in the whole population (n = 7224). Variables included in the model: sex, age, blood pressure, and sugary food. (DOCX 20 kb) [file 12889_2018_5384_MOESM1_ESM.docx]

**Additional file 1**

Table S1. Adjusted ORs for predictors of elevated capillary blood glucose in the whole population (n=7224)

| **Variable** | **Category** | **Adjusted ORs (95% CI)** | **p value** |
| --- | --- | --- | --- |
| Sex | Men | 1.62 (1.31-1.99) | <0.001 |
| Age (years) | < 30 | 1 (reference) |  |
|  | 31-39 | 1.15 (0.85-1.56) | 0.35 |
|  | 40-49 | 1.61 (1.20-2.17) | <0.01 |
|  | ≥50 | 2.07 (1.55-2.78) | <0.001 |
| Blood pressure | Normal | 1 (reference) |  |
|  | High | 1.16 (0.99-1.35) | 0.05 |
| Sugary food | Never / < 3 times per week | 1 (reference) |  |
|  | 3 to 6 times per week | 1.13 (0.96-1.33) | 0.13 |
|  | Once per day or more | 1.20 (0.99-1.44) | 0.05 |
