# Supplementary material for: Predictors of elevated capillary blood glucose in overweight railway French employees: a cross-sectional analysis
Source: BMC Public Health. 2018 Apr 16;18:507. doi: 10.1186/s12889-018-5384-y (PMC5902963; doi:10.1186/s12889-018-5384-y)
Supplement: Supplementary file 2 — Table S2. Multivariate logistic regression model using data from multiple imputation. Description of data: Table S2. presents the results obtained from sensitivity analyses (multiple imputation method) performed to assess the impact of missing data. (DOCX 18 kb) [file 12889_2018_5384_MOESM2_ESM.docx]

Additional file 2

Table S2. Multivariate logistic regression model using data from multiple imputation

| **Variable** | **Category** | **Adjusted ORs (95%CI)** | **p Value** |
| --- | --- | --- | --- |
| Sex | Men | 1.54 (1.28-1.86) | <0.001 |
| Age (years) | < 30 | 1 (reference) |  |
|  | 31-39 | 1.16 (0.87-1.54) | 0.32 |
|  | 40-49 | 1.68 (1.29-2.20) | <0.001 |
|  | ≥50 | 2.13 (1.64-2.78) | <0.001 |
| Blood pressure | Normal | 1 (reference) |  |
|  | High | 1.14 (0.99-1.31) | 0.05 |
| Sugary food | Never / < 3 times per week | 1 (reference) |  |
|  | 3 or 6 times per week | 1.08 (0.93-1.27) | 0.31 |
|  | Once per day or more | 1.15 (0.92-1.43) | 0.21 |
